# Sleep Apnea-Specific Hypoxic Burden and Postoperative Outcomes of Major Noncardiothoracic Surgery

**DOI:** 10.1001/jamanetworkopen.2026.0006

**Published:** 2026-02-24

**Authors:** Sébastien Bailly, Abdelkebir Sabil, Margaux Blanchard, Sandrine Kerbrat, François Goupil, Audrey Thomas, Jérémie Thereaux, Basil Fuchs, Francis Couturaud, Samir Jaber, Dany Jaffuel, Wojciech Trzepizur, Frédéric Gagnadoux

**Affiliations:** 1 Institut de Recherche en Santé Respiratoire des Pays de la Loire, Beaucouzé, France; 2 University Grenoble Alpes, Inserm, CHU Grenoble Alpes, HP2, Grenoble, France; 3 Cloud Sleep Lab, Paris, France; 4 Damad, Plouzane, France; 5 Department of Respiratory Diseases, Le Mans General Hospital, Le Mans, France; 6 Unite de Pathologies Respiratoires, Pole Sante des Olonnes, Olonne sur mer, France; 7 University Brest, CHU Brest, Brest, France; 8 Department of General, Digestive and Metabolic Surgery, La Cavale Blanche University Hospital, Boulevard Tanguy Prigent, Brest, France; 9 Department of Medical Information, La Cavale Blanche University Hospital, Boulevard Tanguy Prigent, Brest, France; 10 Univ Brest, INSERM U1304-GETBO, Brest, France; 11 Department of Pneumology, CHU Brest, Brest, France; 12 Department of Anaesthesiology and Critical Care Medicine B (DAR B), Saint-Eloi Hospital, University Teaching Hospital of Montpellier, PhyMed Exp, Université de Montpellier, INSERM U1046, Montpellier, France; 13 Department of Respiratory Diseases, Montpellier University Hospital, Arnaud de Villeneuve Hospital, Montpellier, France; 14 PhyMed Exp, Université de Montpellier, INSERM U1046 Montpellier, Montpellier, France; 15 Department of Respiratory and Sleep Medicine, Angers University Hospital, Angers, France

## Abstract

**Importance:**

Obstructive sleep apnea (OSA) is a highly prevalent and heterogeneous condition that predisposes to postoperative complications. Adequate metrics of OSA severity to stratify postoperative risk in a clinical setting are needed.

**Objective:**

To evaluate whether the sleep apnea-specific hypoxic burden (SASHB) is associated with postoperative cardiovascular (CV) complications and mortality among patients with OSA undergoing major noncardiothoracic surgery.

**Design, Setting, and Participants:**

Multicenter clinic-based cohort linked with a health administrative database involving adult patients diagnosed with OSA between May 2007 and December 2018 who underwent major noncardiothoracic surgery between OSA diagnosis and December 2024. Data were analyzed from January to December 2025.

**Exposure:**

SASHB defined as the area under the desaturation curve associated with sleep-related obstructive respiratory events.

**Main Outcomes and Measures:**

The primary outcome was a composite of stroke, atrial fibrillation, heart failure, myocardial infarction, venous thromboembolism, and all-cause mortality within 30 days of surgery. The association of SASHB and other OSA severity metrics with the primary outcome was investigated using univariable and multivariable analyses.

**Results:**

Among 2286 patients with OSA (median [IQR] age, 58 [49-66] years; 1472 [64.4%] men) who underwent major noncardiothoracic surgery a median (IQR) of 4.5 (1.9-7.5) years after OSA diagnosis, the primary outcome occurred in 80 patients (3.5%) within 30 days of surgery. The rate of events increased significantly from 1.6% (12 events) in patients with low SASHB (<32% min/h) to 5.8% (44 events) in those with high SASHB (≥80% min/h) at diagnosis. Compared with patients with low SASHB, patients with higher SASHB at diagnosis exhibited increased odds for the primary outcome (adjusted odds ratios, 1.76; 95% CI, 0.86-3.59 and 2.79; 95% CI, 1.42-5.49 for SASHB 32 to <80% and ≥80% min/h, respectively). A risk score based on age, emergency admission before surgery, and SASHB was associated with the primary outcome (area under receiver operating characteristic curve, 0.73; 95% CI, 0.68-0.77). Similar findings were obtained using a simplified version of SASHB automatically derived from the single oximetry signal extracted from diagnostic sleep studies.

**Conclusions and Relevance:**

Among OSA patients undergoing major noncardiothoracic surgery, SASHB was significantly associated with the risk of 30-day postoperative mortality and CV complications. Further research is needed to determine whether interventions guided by SASHB scores can modify postoperative risk in patients with OSA.

## Key Points

**Question:**

Is there an association between the sleep apnea–specific hypoxic burden (SASHB) and 30-day postoperative mortality and cardiovascular (CV) morbidity among patients with obstructive sleep apnea (OSA) undergoing major noncardiothoracic surgery?

**Findings:**

In this cohort study that included 2286 patients with OSA undergoing major noncardiothoracic surgery, the rate of a composite outcome of postoperative complications (all-cause mortality, stroke, atrial fibrillation, heart failure, myocardial infarction, and venous thrombo-embolism) increased significantly from 1.6% in patients with low SASHB (<32% min/h) to 5.8% in those with high SASHB (≥80% min/h) at diagnosis.

**Meaning:**

These findings suggest that among OSA patients undergoing major noncardiothoracic surgery, SASHB was significantly associated with the risk of 30-day postoperative mortality and CV complications.

## Teaser

This cohort study evaluates whether the sleep apnea-specific hypoxic burden (SASHB), a method for quantifying quantifying obstructive sleep apnea, is associated with postoperative cardiovascular complications and mortality among patients undergoing major noncardiothoracic surgery.

## Introduction

Obstructive sleep apnea (OSA) is a prevalent disease characterized by recurrent episodes of obstruction of the upper airways, resulting in intermittent hypoxemia and sleep fragmentation, with potential repercussions on cardiovascular health.^1,2,3^ Anatomical and physiological factors predisposing patients to OSA increase vulnerability to airway obstruction under general anesthetics, sedatives, and postoperative analgesics that relax the upper airway muscles and impair ventilatory response. Although these phenomena could predispose patients with OSA to postoperative mortality complications, recent analyses of large database repositories showed conflicting results.^4,5,6^

Given its estimated prevalence,^7^ risk stratification in patients with OSA undergoing surgery is an important challenge. There is a growing awareness that traditional metrics of OSA severity such as the apnea-hypopnea index (AHI) do not adequately capture the physiological consequences of upper airway obstructions and the susceptibility to adverse cardiovascular outcomes.^3,8^ The failure to consider OSA heterogeneity might have contributed to the null findings of trials with cardiovascular end points in OSA.^9,10,11^ Significant efforts have been made to better capture the heterogeneity of OSA by developing biomarkers of cardiovascular vulnerability derived from sleep studies.^3,8,12^ The sleep apnea-specific hypoxic burden (SASHB) describes both the frequency of upper airway obstructions during sleep, as well as the duration and depth of related oxygen desaturations.^13^ Data from several clinic and population-based cohorts have shown that SASHB is more strongly associated with cardiovascular outcomes than traditional metrics of OSA severity.^14,15,16,17,18^ Furthermore, a simplified version of SASHB derived from pulse oximetry (HB_oxi_) was demonstrated to better identify OSA patients with cardiovascular benefit from PAP therapy than traditional metrics of OSA severity, supporting its clinical relevance for risk stratification and therapeutic decision-making.^19,20^ Based on previous studies demonstrating the relevance of oxygen desaturation metrics to the relationship between OSA and postoperative outcomes,^5,21,22,23,24,25^ we hypothesized that SASHB could help identify OSA patients at high risk of postoperative complications. The objective of the present study was to evaluate, in a large multicenter cohort of patients diagnosed with OSA, the association between SASHB derived from baseline sleep study, and a composite of all-cause deaths and cardiovascular events within 30 days of major noncardiothoracic surgery.

## Methods

### Study Design and Population

The study relied on data collected by the multicenter Pays de la Loire Sleep Cohort, further linked with data from the French administrative health care database (SNDS; see eAppendix in Supplement 1).^17,26,27,28^ Adult patients diagnosed with OSA between May 15, 2007, and December 31, 2018, who underwent elective or emergency major noncardiothoracic surgery between the diagnostic sleep study and December 31, 2024, were eligible for the study. Approval was obtained from the University of Angers ethics committee and the Comité Consultatif sur le Traitement de l’Information en matière de Recherche dans le domaine de la Santé. All patients had given their written informed consent.

### Procedures

The diagnosis of OSA was based on polysomnography (CID102L8D, Cidelec) or home sleep apnea testing (CID102L, Cidelec), according to pretest clinical probability. Relevant metrics of OSA severity included the AHI (events per hour of sleep or recording), the 3% oxygen desaturation index (events per hour), the percentage of sleep or recording time with oxygen saturation less than 90%, and the SASHB. As described previously,^17^ SASHB was defined as the area under the desaturation curve associated with respiratory events, calculated over a participant-specific search window for each event. The SASHB was then obtained by adding these individual desaturation areas and dividing the total sleep (or recording) time, with the units of SASHB being % min/h. A simplified version of SASHB automatically derived from the single oximetry signal extracted from diagnostic sleep studies, HB_oxi_, was also calculated. As described previously, HB_oxi_ was defined as the total area under all desaturation curves divided by the total recording time.^20^ Each patient completed surveys including anthropometric data, socioeconomic status, medical history, and the Epworth sleepiness scale.^29,30^

As previously described,^31^ positive airway pressure (PAP) therapy was prescribed to patients with severe OSA (AHI ≥30) or with moderate OSA (AHI 15 to <30) with cardiovascular comorbidities or severe daytime sleepiness. Based on the digital downloads from PAP devices, the mean of all semiannually recorded measurements of daily PAP use was then calculated over the follow-up period. Patients who had not discontinued PAP and used it a mean of 4 hours or more per night during the follow-up period were assigned to the PAP adherent group. Patients who stopped the use of PAP or those who used the device a mean of less than 4 hours per night constituted the nonadherent group.^32^

### End Point

The primary end point was a composite of stroke, atrial fibrillation, heart failure, myocardial infarction, venous thromboembolism, and all-cause mortality within 30 days of surgery. Complications were identified using specific *International Statistical Classification of Diseases and Related Health Problems, Tenth Revision* codes obtained from the SNDS database (eTable 1 in Supplement 1).

### Statistical Analysis

Data were described using numbers and percentages for qualitative variables and median and IQR for quantitative variables. Comparisons between groups (with and without primary outcome) were performed using the χ^2^ test for qualitative variables and the Mann-Whitney test for quantitative variables. For variables included in multivariable models, missing continuous values were replaced by the cohort median and missing binary values by the most frequent category. Variables with higher missingness were not imputed as they were used only descriptively. Three nested logistic regression models were performed to identify the most informative metrics of OSA severity associated with the primary outcome: a null model, a model with variables selected using a stepwise selection method, and a full model including all variables significant in the univariable analysis. Due to collinearity, according to Pearson correlations, metrics of OSA severity were considered separately in each model and entered as continuous variables. Finally, the model with the lowest Akaike information criterion (AIC) was selected. An elastic net regression was performed including all significant metrics of OSA severity to confirm the variable selection. Further analyses categorizing SASHB scores into tertiles were performed to increase the clinical interpretability of graphical presentations. Prespecified subgroup analyses were conducted by age (<58 vs ≥58 years), sex, with and without a history of cardiovascular disease, type of sleep recording (polysomnography vs home sleep apnea testing), duration between sleep study and surgery date (<4.5 vs ≥4.5 years), PAP status (adherent vs no PAP or PAP nonadherent). Interaction *P* values between subgroups and SASHB tertiles were computed with a significance level of <.05. We additionally performed a descriptive analysis of baseline patient characteristics according to PAP status.

The final step was to propose a threshold of SASHB and its simplified version HB_oxi_ to estimate risk of 30-day postoperative complications. The dataset was divided into a training dataset (1601 patients [70%]; 56 events) and a test dataset (685 patients [30%]; 24 events) with stratification on outcome to ensure that the prevalence was maintained between the 2 datasets. Because the number of events in the training set was low, we applied simple random oversampling of the minority class (sampling with replacement) to increase the number of events from 56 to 184. Oversampling was used only in the training dataset, and inverse-probability weights were applied in the logistic regression model to restore the original event proportion. Variable selection was confirmed in the training dataset to identify the most parsimonious set of variables to estimate the likelihood of an event. A bootstrap of 1000 iterations was performed with a stepwise selection process. Variables selected in more than 50% of the iterations were included in the final model. At the end of the bootstrapping process, 3 variables were retained: age, emergency admission before surgery events, and SASHB (or HB_oxi_). The receiver operating characteristic area under the curve (ROC AUC) of the final model was computed with its 95% CI.

A logistic regression model was fitted to the oversampled dataset. Weighting was applied to account for the imbalance between groups due to oversampling. From the final logistic regression model, a score was proposed based on the estimated parameter of the logistic regression. The score was finally applied to the test set to define the performance of the score (sensitivity, specificity, positive [PPV] and negative [NPV] predictive values, and Youden index). Statistical analyses were performed using SAS version 9.4 (SAS Institute Inc) from January to December 2025.

## Results

### Population Description

**Figure 1.**
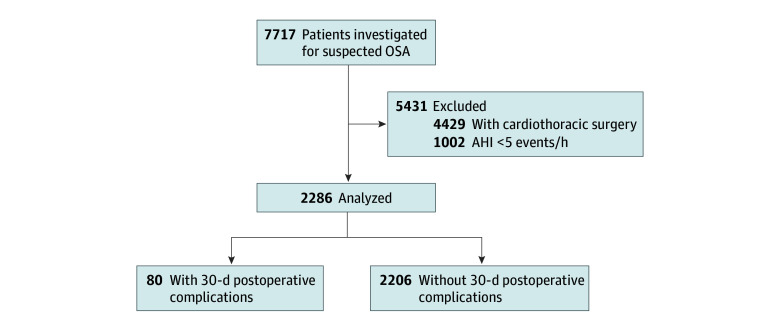
Flowchart of the Study AHI indicates apnea-hypopnea index; OSA, obstructive sleep apnea.

Of 7717 patients from the cohort, 2286 patients with a confirmed diagnosis of OSA (AHI ≥5 events per hour) who underwent major noncardiothoracic surgery with a median (IQR) of 4.5 (1.9-7.5) years after the diagnostic sleep study had their data analyzed (Figure 1). Patient characteristics are detailed in Table 1. Patients were predominantly male (1472 patients [64%]), with a median (IQR) age of 58 (49-66) years. As shown in Table 2, the most common surgical procedures were orthopedic (803 patients [36.1%]) and digestive (803 patients [36.1%]) procedures. For 241 patients (10%), surgery was considered urgent, as it was performed shortly after admission to the emergency department. As shown in eTable 2 in Supplement 1, PAP-adherent patients had more severe OSA at baseline.

**Table 1.** Patient Characteristics

Variable	Patients, No. (%)				*P* value
	All patients (N = 2286)	No postoperative complication (n = 2206)	Postoperative complication (n = 80)	Missing	
Age, median (IQR), y	58 (49-66)	58 (48-66)	63.5 (55.5-72)	0	<.001
Sex					
Female	814 (35.6)	793 (35.9)	21 (26.2)	0	.08
Male	1472 (64.4)	1413 (64.1)	59 (73.8)		
BMI, median (IQR)^a^	30.4 (26.7-35.0)	30.3 (26.7-35)	31.2 (25.8-35.7)	0	.50
Daily alcohol intake	645 (34.8)	615 (34.3)	30 (50.8)	435	.01
Smoking status					
Former smokers	899 (38.5)	866 (39.3)	33 (41.3)	0	.66
Current smokers	401 (17.5)	390 (17.7)	11 (13.8)		
Nonsmokers	986 (43.1)	950 (43.1)	36 (45.0)		
Living in couple	1853 (81.1)	1791 (81.2)	62 (77.5)	0	.41
Professional status					
Active	941 (43.5)	922 (44.1)	19 (26.0)	110	.01
Inactive	298 (13.8)	286 (13.7)	12 (16.4)		
Retired	926 (42.8)	884 (42.3)	42 (57.5)		
Professional category					
Farmer	88 (4.6)	84 (4.5)	4 (5.9)	320	.31
Craftsman	208 (10.9)	199 (10.8)	9 (13.2)		
Executive	324 (16.9)	309 (16.7)	15 (22.1)		
Intermediate	387 (20.2)	379 (20.5)	8 (11.8)		
Employee	391 (20.4)	376 (20.3)	15 (22.1)		
Workers	518 (27.0)	501 (27.1)	17 (25.0)		
Epworth score, median (IQR)	10.0 (6.0-13.0)	10.0 (6.0-14.0)	8.0 (5.0-13.0)	0	.05
Medical history					
CVD	396 (17.3)	370 (16.8)	26 (32.5)	0	<.001
Stroke	137 (6.3)	128 (6.1)	9 (12.0)	124	.04
AF	103 (4.8)	99 (4.8)	4 (5.3)	139	.85
Heart failure	119 (5.5)	104 (5.0)	15 (20.0)	133	<.001
CAD	181 (8.3)	170 (8.1)	11 (14.1)	85	.06
Hypertension	971 (42.5)	930 (42.2)	41 (51.3)	0	.11
Diabetes	418 (18.3)	396 (18.0)	22 (27.5)	0	.03
COPD	305 (13.3)	291 (13.2)	14 (17.5)	0	.27
Baseline sleep study					
PSG	1101 (48.2)	1074 (48.7)	27 (33.8)	0	.01
HSAT	1185 (51.8)	1132 (51.3)	53 (66.3)		
AHI, median (IQR), events/h	30.0 (16.0-44.0)	30.0 (15.0-44.0)	42.5 (22.5-60.0)	0	<.001
ODI, median (IQR), events/h	22.0 (11.0-39.0)	21.6 (11.0-38.0)	35.5 (18.5-56.5)	1	<.001
T90, median (IQR), %	4.0 (0.0-17.0)	4.0 (0.0-17.0)	12.5 (1.0-32.0)	0	<.001
SASHB, median (IQR)	51.8 (24.0-99.8)	51.1 (23.5-98.0)	86 (47-177.8)	0	<.001
HB_oxi_^b^	56.3 (30.8-104)	55.5 (30.4-102.4)	87.5 (47.0-180.1)	0	<.001
Medication in the year before surgery					
Muscle relaxants	165 (7.2)	161 (7.3)	4 (5.0)	0	.44
Antidepressant drugs	545 (23.8)	525 (23.8)	20 (25.0)		.80
Anxiolytics	706 (30.9)	683 (31.0)	23 (28.8)		.67
PAP status					
No PAP	982 (43.0)	955 (43.3)	27 (33.8)	0	.02
Nonadherent	576 (25.2)	560 (25.4)	16 (20.0)		
Adherent	728 (31.8)	691 (31.3)	37 (46.3)		

Abbreviations: AF, atrial fibrillation; AHI, apnea-hypopnea index; BMI, body mass index; CAD, coronary heart disease; COPD, chronic obstructive pulmonary diseases; CVD, cardiovascular diseases; HSAT, home sleep apnea testing; ODI, 3% oxygen desaturation index; PAP, positive airway pressure therapy; PSG, polysomnography; SASHB, sleep apnea-specific hypoxic burden; T90, % of sleep (or recording) time with peripheral oxygen saturation less than 90%.

^a^ BMI is calculated as weight in kilograms divided by height in meters squared.

^b^ Simplified version of SASHB automatically derived from the single oximetry signal extracted from diagnostic sleep studies.

**Table 2.** Noncardiothoracic Surgical Procedures

Variable	Patients, No. (%)			*P* value
	All patients (N = 2286)	No postoperative complication (n = 2206)	Postoperative complications (n = 80)	
Type of surgery				
Orthopedic	803 (35.1)	783 (35.5)	20 (25)	.002
Digestive	803 (35.1)	780 (35.4)	23 (28.8)	
Neurological	286 (12.5)	266 (12.1)	20 (25)	
Other^a^	394 (17.2)	377 (17.1)	17 (21.3)	
Emergency admission before surgery	241 (10.5)	214 (9.7)	27 (33.7)	<.001
Delay between sleep study and surgery, median (IQR), d	1662 (714-2742)	1659 (717-2743)	1753 (694-2692)	.98
History of surgery	122 (5.3)	117 (5.3)	5 (6.3)	.71

^a^ Other surgery included gynecology (95 patients); ear, nose, and throat (3 patients); urology (272 patients); and internal (24 patients).

### Outcomes

The primary outcome occurred in 80 patients (3.5%) and included atrial fibrillation in 29, death in 18, stroke in 14, heart failure in 12, myocardial infarction in 7, and venous thromboembolism in 12 patients. An emergency admission before surgery was noted in 33.7% of patients with postoperative complication vs 9.7% of patients with no postoperative complication (χ^2^_1_ *=* 47.34; *P* < .001). The majority of complications (53 patients [66%]) occurred on the first day after surgery (eTable 3 in Supplement 1). Patients with postoperative complications were older, more frequently male, had more frequent comorbidities, and exhibited greater OSA severity (Table 1).

Logistic regression models assessing the association between metrics of OSA severity and the primary outcome are presented in eTable 4 in Supplement 1 and Table 3. All 5 metrics (considered continuous values) were significantly associated with the occurrence of the primary outcome in the 3 consecutive models (except for the time with oxygen saturation <90% in model 3), with the lowest AIC values being observed for SASHB for each of the 3 models. The stronger association with the primary outcome of SASHB and its simplified version, HB_oxi_, compared with traditional metrics of OSA severity, was confirmed by elastic net regression analysis.

**Table 3.** Logistic Regression Models Assessing the Association Between Metrics of Obstructive Sleep Apnea Severity and the Primary Outcome

Model	OR (95% CI)	*P* value	AIC
Model 1^a^			
AHI	1.02 (1.01-1.03)	<.001	682.5
ODI	1.02 (1.01-1.03)	<.001	680.0
T90	1.01 (1.01-1.02)	.003	689.8
SASHB	1.00 (1.00-1.01)	<.001	674.1
HB_oxi_^b^	1.00 (1.00-1.01)	<.001	676.7
Model 2^c^			
AHI	1.02 (1.01-1.03)	.01	639.3
ODI	1.02 (1.01-1.03)	<.001	638.5
T90	1.01 (0.99-1.02)	.09	647.5
SASHB	1.00 (1.00-1.01)	<.001	632.2
HB_oxi_	1.00 (1.00-1.01)	<.001	633.7
Model 3^d^			
AHI	1.02 (1.01-1.03)	.001	645.4
ODI	1.02 (1.01-1.03)	.001	644.5
T90	1.01 (0.99-1.02)	.09	652.2
SASHB	1.00 (1.00-1.01)	<.001	639.0
HB_oxi_	1.00 (1.00-1.01)	<.001	640.2

Abbreviations: AHI, apnea-hypopnea index; AIC, Akaike information criterion; ODI, 3% oxygen desaturation index; OR, odds ratio; SASHB, sleep apnea-specific hypoxic burden; T90, % of sleep (or recording) time with peripheral oxygen saturation less than 90%.

^a^ Model 1: unadjusted.

^b^ Simplified version of SASHB automatically derived from the single oximetry signal extracted from diagnostic sleep studies.

^c^ Model 2: all variables were introduced in the model and were automatically selected using a stepwise selection method. The final model 2 included age and emergency admission before surgery.

^d^ Model 3: adjusted for age, sex, hypertension, history of cardiovascular events, tobacco consumption, chronic obstructive pulmonary disease, and emergency admission before surgery.

**Figure 2.**
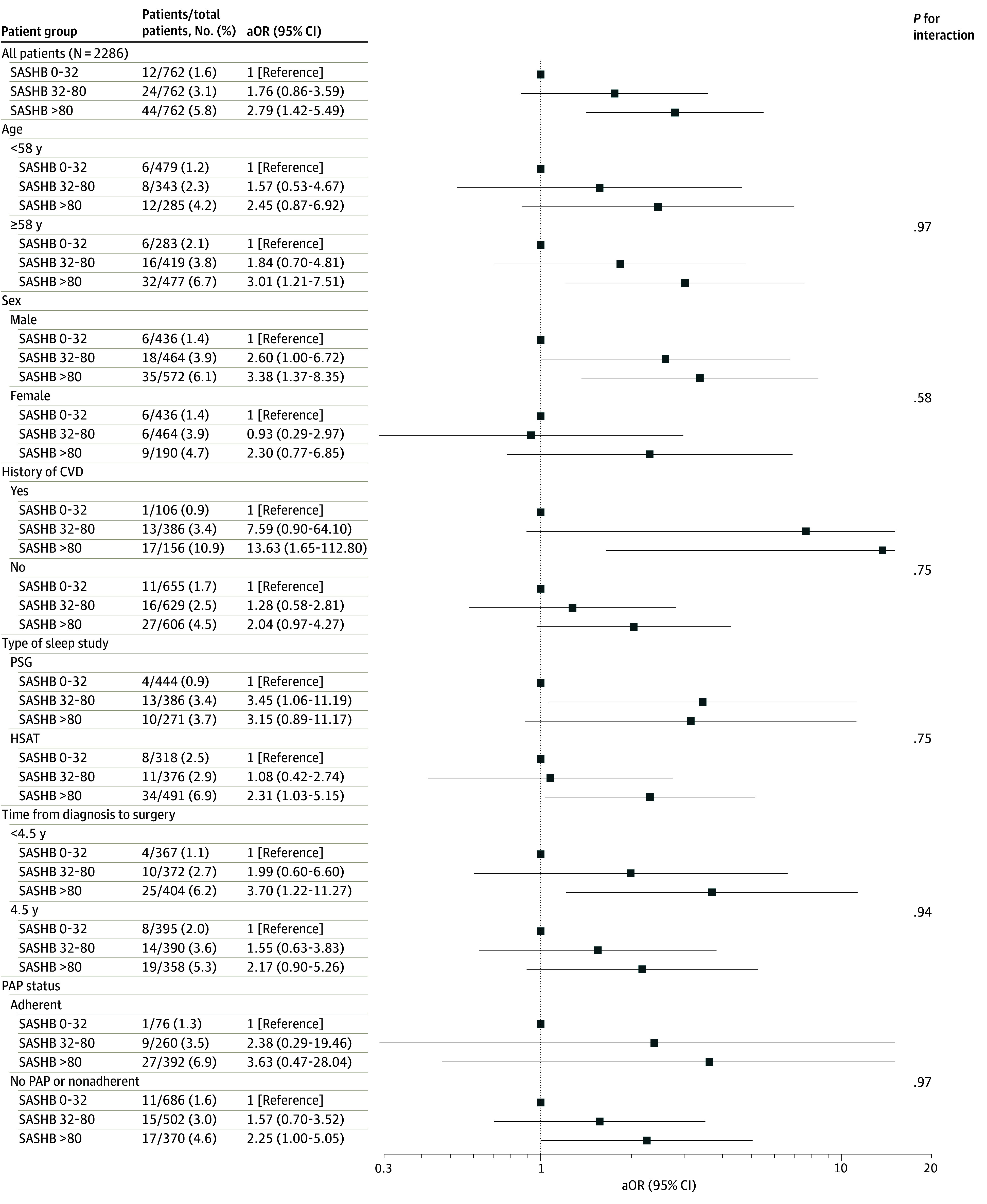
Forest Plot of Association of Sleep Apnea-Specific Hypoxic Burden (SASHB) With the Primary Composite Outcome Within 30 Days of Surgery, in the Entire Population and in Patient Subgroups Primary composite outcome was all-cause mortality, stroke, atrial fibrillation, heart failure, myocardial infarction, and venous thrombo-embolism within 30 days of surgery. Models were adjusted for age, sex, history of CVD, chronic obstructive pulmonary disease, tobacco consumption, and emergency admission before surgery. aOR indicates adjusted odds ratio; CVD, cardiovascular diseases; HSAT, home sleep apnea testing; PAP, positive airway pressure therapy; PSG, polysomnography.

For interpretability, Figure 2 and eTable 5 in Supplement 1 present odds ratios (ORs) from multivariable logistic regression models assessing the association of SASHB categorized in tertiles of the variable and the primary outcome. Compared with patients with SASHB less than 32% min/h, patients with higher SASHB at diagnosis exhibited increased odds for the primary outcome (adjusted OR, 1.76; 95% CI, 0.86-3.59; and OR, 2.79; 95% CI, 1.42-5.49 for SASHB 32 to <80% and ≥80% min/h, respectively) in the entire population. Consistently, the absolute event rate increased significantly from 1.6% (12 events) in patients with low SASHB to 5.8% (44 events) in those with high SASHB at diagnosis. The association between SASHB and the primary outcome was similar across patient subgroups based on demographics, history of cardiovascular diseases, type of sleep study, delay between OSA diagnosis and surgery, and PAP status.

### Risk Score for 30-Day Postoperative Complications

A risk scoring model incorporating age, emergency admission before surgery, and SASHB was developed and validated. The final logistic regression provided the following estimation for the logistic regression parameters: 0.039 (±0.0119) for age as continuous variable, 1.67 (±0.30) for emergency admission before surgery, and 0022 (±0.001) for SASHB as continuous variable. The ROC-AUC of the final model was 0.73 (95% CI, 0.68-0.77) and was similar for HB_oxi_ (0.75; 95% CI, 0.67-0.78).

The proposed score (age × 0.039 + emergency admission × 1.67 + SASHB × 0.0022) with a range from 1 to 6 was applied to the training dataset and rounded to the unit. In the test dataset, we computed the sensitivity and the specificity for each value of the threshold to identify the score that maximized the sensitivity, the one that maximized the specificity, and the one that maximized the Youden index (Se + Sp - 1). Because postoperative complications were rare in the test dataset (80 patients [3.5%]), PPV remained low across all thresholds (4% to 20%), whereas NPV was consistently high (≥96%), as expected due to the low primary outcome prevalence. In the validation dataset, a score threshold of 3 maximized the Youden index, providing the best compromise between sensitivity (0.56; SE, 0.037) and specificity (0.77; SE, 0.016) for estimating the risk of postoperative complications. These performances were similar for HB_oxi_.

## Discussion

In this cohort study of adults with OSA undergoing major noncardiothoracic surgery, OSA severity assessed by the SASHB was significantly associated with a risk of 30-day postoperative cardiovascular complications and mortality. A risk score based on age, emergency admission before surgery and SASHB was associated with the primary outcome. Similar results were obtained with the simplified version of SASHB automatically derived from the single oximetry, HB_oxi_.

To date, the association of OSA with an increase in major postoperative cardiovascular events remains a subject of debate.^6^ In a retrospective cohort analysis^33^ including 1 813 974 surgical patients, those with OSA (10.2%) had higher rates of atrial fibrillation and congestive heart failure but a lower rate of myocardial infarction, lower mortality, and shorter length of hospital stay. There is increasing evidence that AHI provides an imperfect assessment of the risk of postoperative complications, and that parameters of sleep-related hypoxia may provide additional value in risk stratification and minimization.^4,22,23,24,25^ Among 1218 patients undergoing major noncardiac surgery, oxygen desaturation index of 30 or more events per hour and a time spent with oxygen saturation below 80% for 10 or more minutes were independently associated with of 30-day postoperative cardiovascular events.^24^ In a cohort of 6770 consecutive patients who underwent a procedure involving general anesthesia, the time with oxygen desaturation below 90% was the only factor independently associated with postoperative cardiorespiratory complications within 30 days, which occurred in 5.3% of participants.^4^ However, the time spent with oxygen saturation below 90% characterizes not only OSA-related intermittent hypoxemia, but also sustained hypoxemia consecutive to comorbid conditions such as chronic obstructive pulmonary disease or obesity.^34,35^

To our knowledge, our study is the first to have evaluated the value of SASHB with regard to estimating postoperative risk. Our finding that high SASHB is an independent factor associated with risk for postoperative mortality and cardiovascular complications is consistent with previous analyses in nonsurgical settings.^14,15,16,17,18^ The association of SASHB with the primary outcome was significant regardless of the time between sleep study and surgery, highlighting the robustness of SASHB over time. Given the global burden of OSA,^7^ the high cost and limited availability of in-laboratory polysomnography, home sleep apnea testing has emerged as a more accessible OSA diagnostic test.^36^ In our study, SASHB was associated with the primary outcome regardless of the sleep recording type used for OSA diagnosis. Altogether, these findings suggest that SASHB derived from a simple OSA diagnostic test, combined with common risk factors such as age and emergency admission before surgery, could contribute to a better estimate of the risk of postoperative complications. Because postoperative complications were rare in our cohort, the NPV of the score was consistently high. This finding suggests that the score performs best as a rule-out tool, which might be clinically useful for identifying individuals who may safely undergo standard postoperative monitoring, thereby helping to prioritize resources for higher-risk patients.

At present, there is a lack of robust evidence for the perioperative effectiveness of PAP therapy due to ethical obstacles associated with randomizing this therapy.^6^ In clinical settings, it has been reported that less than 20% of patients with OSA undergoing total hip and knee arthroplasties receive PAP therapy in the perioperative phase.^37^ A recent systematic review and meta-analysis^38^ estimated that PAP therapy was associated with a 28% reduction in respiratory complications and 56% reduction in unplanned ICU admission after noncardiac surgery. In OSA patients undergoing cardiac surgery, PAP therapy was associated with a 37% reduction in postoperative cardiac complications. Interestingly, we found that higher SASHB values were significantly associated with the primary outcome in nontreated or PAP nonadherent patients, but not in PAP adherent participants. However, a formal test for interaction was not significant. Considering differences in sample sizes and baseline patient characteristics according to PAP status and residual confounding by the healthy adherer effect,^39,40^ this finding should not be interpreted as the demonstration of a preventive effect of PAP therapy against postoperative complications. Several strategies likely to reduce SASHB and mitigate its impact on the risk of postoperative complications warrant evaluation in future studies, including support programs on PAP adherence combined with weight loss interventions effective in improving SASHB,^41^ optimized intraoperative and perioperative management with structured PAP protocols or prolonged supplemental oxygen therapy, and regional analgesia or avoidance of postoperative opioids. Additional research is also needed to investigate whether continuous pulse oximetry monitoring would suffice as safe and cost-effective postoperative monitoring in OSA patients with high SASHB.^42^

### Limitations

This study has limitations. Relying on administrative health databases for outcomes may introduce misclassification biases in outcome ascertainment. The observational study design limits causal inference, and residual confounding factors by unmeasured variables, such as detailed intraoperative anesthetic practices and nuanced postoperative management differences, cannot be excluded. In particular, we were unable to assess the consequences of opioid or oral morphine equivalents usage in perioperative or postoperative periods on OSA physiology and postoperative risk, as these data were not captured in our dataset. In contrast to the current guideline recommendations,^43^ recent studies showed no change in postoperative outcomes with regional vs general analgesia or avoidance of postoperative opioids.^5,44^ Furthermore, we lacked precise data on immediate PAP use in the recovery room and during the 30 postoperative days, which could impact outcomes in certain patients.^45^ We also acknowledge that the accuracy of the estimate is limited by the small number of postoperative events. This leads to statistically significant associations with a degree of uncertainty. Further large-scale cohort studies will be needed to confirm the effect size and direction of the observed associations.^46^ Although it has been shown to overcome some of the limitations of less quantitative OSA severity markers, SASHB as well as other OSA-specific intermittent hypoxemia measures have some limitations, including the current lack of population-specific threshold values with prognostic relevance.^47^ Further studies are required to define a specific actionable SASHB threshold for perioperative PAP protocols and enhanced monitoring. Additionally, the SASHB used in our main analysis was derived from previous manually scored sleep studies. Currently, OSA remains underdiagnosed in the surgical population due to the limited accessibility of in-laboratory sleep recordings. Overnight pulse oximetry is easy to use in the ambulatory setting, and has emerged as an alternative preoperative screening tool for OSA, which circumvents the barriers associated with polysomnography.^24^ In our additional analysis, we demonstrated that HB_oxi_ automatically derived from pulse oximetry had similar performance to that of SASHB derived from manually scored sleep studies, which may enhance the accessibility of this biomarker through simple preoperative oximetry.

## Conclusions

Among OSA patients undergoing major noncardiothoracic surgery, SASHB was significantly associated with the risk of 30-day postoperative mortality and cardiovascular complications. Further research is needed to determine whether interventions guided by SASHB scores can modify postoperative risk in patients with OSA.

## Notes

Supplement 1. eAppendix. Details on the Pays de la Loire Sleep Cohort (PLSC) and the Linkage Process With the French Administrative Health Care Database eTable 1. Procedure Codes eTable 2. Baseline Patient Characteristics According to PAP Status (Adherent vs no PAP or PAP Nonadherent) eTable 3. Duration Between Surgery and Complication Occurrence eTable 4. Univariable Logistic Regression Analyses of Factors Associated With the Primary Outcome eTable 5. Association of Sleep Apnea-Specific Hypoxic Burden (SASHB) with the Primary Composite Outcome (All-Cause Mortality, Stroke, Atrial Fibrillation, Heart Failure, Myocardial Infarction, and Venous Thrombo-Embolism) Within 30 Days of Surgery, in the Entire Population and in Patient Subgroups

Supplement 2. Data Sharing Statement
