# Supplementary material for: Sleep Apnea-Specific Hypoxic Burden and Postoperative Outcomes of Major Noncardiothoracic Surgery
Source: JAMA Netw Open. 2026 Feb 24;9(2):e260006. doi: 10.1001/jamanetworkopen.2026.0006 (PMC12933281; doi:10.1001/jamanetworkopen.2026.0006)
Supplement: Supplement 1. — eAppendix. Details on the Pays de la Loire Sleep Cohort (PLSC) and the Linkage Process With the French Administrative Health Care Database eTable 1. Procedure Codes eTable 2. Baseline Patient Characteristics According to PAP Status (Adherent vs no PAP or PAP Nonadherent) eTable 3. Duration Between Surgery and Complication Occurrence eTable 4. Univariable Logistic Regression Analyses of Factors Associated With the Primary Outcome eTable 5. Association of Sleep Apnea-Specific Hypoxic Burden (SASHB) with the Primary Composite Outcome (All-Cause Mortality, Stroke, Atrial Fibrillation, Heart Failure, Myocardial Infarction, and Venous Thrombo-Embolism) Within 30 Days of Surgery, in the Entire Population and in Patient Subgroups [file jamanetwopen-e260006-s001.pdf]

Supplemental Online Content

Bailly S, Sabil A, Blanchard M, et al. Sleep apnea-specific hypoxic burden and postoperative outcomes of major noncardiothoracic surgery. *JAMA Netw Open*. 2026;9(2):e260006. doi:10.1001/jamanetworkopen.2026.0006

eAppendix. Details on the Pays de la Loire Sleep Cohort (PLSC) and the Linkage Process With the French Administrative Health Care Database

eTable 1. Procedure Codes

eTable 2. Baseline Patient Characteristics According to PAP Status (Adherent vs no PAP or PAP Nonadherent)

eTable 3. Duration Between Surgery and Complication Occurrence

eTable 4. Univariable Logistic Regression Analyses of Factors Associated With the Primary Outcome

eTable 5. Association of Sleep Apnea-Specific Hypoxic Burden (SASHB) with the Primary Composite Outcome (All-Cause Mortality, Stroke, Atrial Fibrillation, Heart Failure, Myocardial Infarction, and Venous Thrombo-Embolism) Within 30 Days of Surgery, in the Entire Population and in Patient Subgroups

This supplemental material has been provided by the authors to give readers additional information about their work.

© 2026 Bailly S et al. *JAMA Network Open*.

eAppendix. Details on *the Pays de la Loire Sleep Cohort (PLSC)* and the Linkage Process With the French Administrative Health Care Database

Since May 15, 2007, consecutive patients ≥ 18 years investigated for suspected obstructive sleep apnea (OSA) in 7 centers from the Pays de la Loire were eligible for inclusion in the *PLSC*. Patients with a high clinical probability of OSA were investigated by home sleep apnea testing (HSAT), those with a low likelihood of OSA and/or coexisting sleep disorders were diagnosed with in-lab polysomnography (PSG) (CID102LTM and CID102L8DTM respectively; CIDELEC, France), using recommended scoring rules. Each patient enrolled in the *PLSC* completed questionnaires and surveys including anthropometric data, smoking habits, alcohol consumption, medical history, and medication use. Patients with learning difficulties, who were unable to fill in the questionnaires, or read and/or speak French, and patients with neuromuscular diseases or chronic respiratory failure were not included in *PLSC*. Approval was obtained from the University of Angers Ethics Committee and the 'Comité Consultatif sur le Traitement de l'Information en matière de Recherche dans le domaine de la Santé' (CCTIRS; 07.207bis). The database is anonymous and its linkage with the French administrative health care database (SNDS) database complied with the restrictive requirements of the 'Commission Nationale Informatique et Liberté' (CNIL), the French information technology, and personal data protection authority. Specific approval was obtained from the CNIL to perform this study. The *PLSC* data manager submitted personal identifiers (gender, date of birth [month/year], date and location where overnight PSG or HSAT was performed, and residency postcode) to the National Health Insurance Fund (CNAM), alongside a pseudo-anonymized patient record identifier (link ID) for all patients in their dataset. Pseudo-anonymized identifiers (NUM_ENQ) in the SNDS file were matched with pseudo-anonymized (link ID) identifiers from the *PLSC*, through an iterative deterministic method comprising a series of progressively less restrictive steps, generated from combinations of gender, date of birth (year and month), date (+/- 3 days) and location (hospital identification) where overnight PSG or HSAT was performed, and residency postcode. Overnight PSG or HSAT were identified in the 'Programme de Médicalisation des Systèmes d'Information' (PMSI) database through six codes for medical acts

© 2026 Bailly S et al. *JAMA Network Open*.

(AMQP010-015). Records matched at a given step were not available for matching in subsequent steps. Steps included relaxation on codes for sleep recordings, which were no longer, required. The CNAM generated a linker file. The linker file contains a pair of pseudo-anonymized identifiers (NUM_ENQ, link ID) for each linked patient that was used to merge the *PLSC* dataset with the dataset extracted from SNDS.

© 2026 Bailly S et al. *JAMA Network Open*.

Postoperative complications

The algorithm to identify the first occurrence of CV events or death was based on data from the SNDS, which links anonymously information for all health care insurance reimbursement claims to the national hospital discharge database (PMSI). The PMSI provides detailed medical information about all stays in public and private hospitals, including main and associated discharge diagnosis ICD-10 codes. Cardiovascular complication was defined if the event is observed after the date of surgery and before day 30 for one of the following events:

- Myocardial infarction: I200, I200+0, I21, I22, or I23; or the date of a hospitalization during which a coronary revascularization procedure (angioplasty or bypass) was performed.
- Stroke: the first hospital stay with a main discharge diagnosis G45, G46, I60-I64, or I69 or the entry date of the first hospital stay with same associated diagnosis.
- Heart failure was defined as the date of entry of the first hospital stay with a principal or associated diagnosis of I50, I110, or I13.
- Venous thrombo-embolism: I26 et I80.0-3
- Deaths from any cause: were identified through a link with the Social Security Administration Death Master File.

eTable 1. Procedure Codes

eTable 1a: Codes for exclusion of cardiovascular surgery

Homogeneous patient groups' codes from SNDS were considered to identify surgery interventions.

Cardiovascular surgery interventions were excluded from the analysis

01C061, 01C062, 01C063	Interventions on the precerebral vascular system
05C02Z	Valve replacement surgery with extracorporeal circulation
05C022, 05C023, 05C024	Valve replacement surgery with extracorporeal circulation and cardiac catheterization or coronary angiography
05C031, 05C032, 05C033, 05C034	Valve replacement surgery with extracorporeal circulation, without cardiac catheterization or coronary angiography
05C042, 05C043	Coronary artery bypass grafting with cardiac catheterization or coronary angiography
05C051, 05C052, 05C053, 05C054	Coronary artery bypass grafting with cardiac catheterization or coronary angiography

05C062, 05C063, 05C064	Other cardiothoracic procedures, with extracorporeal circulation
05C081, 05C082, 05C083, 05C084	Other cardiothoracic procedures, without extracorporeal circulation
05C101, 05C102, 05C103, 05C104, 05C10W	Major revascularization surgery
05C111, 05C112, 05C113, 05C114	Other vascular surgery procedures

eTable 1b: Codes for digestive surgeries

06C031, 06C032, 06C033, 06C034	Rectal resections
06C041, 06C042, 06C043, 06C044, 06C04V, 06C04W	Major surgery on the small intestine and colon
06C071, 06C072, 06C073	Minor surgery on the small intestine and colon
06C081, 06C082, 06C083, 06C084	Complicated appendectomies
06C091, 06C092, 06C093	Uncomplicated appendectomies
06C111, 06C112, 06C113, 06C11J, 06C11V	Reconstructive surgery for hernias and eventrations excluding inguinal and crural hernias
06C11W, 06C121, 06C122, 06C124, 06C12J, 06C12V	Repair of inguinal and crural hernias
06C131, 06C132, 06C133	Release of peritoneal adhesions
06C141, 06C142, 06C143, 06C14V	Rectal and anal procedures other than rectal resections
06C151, 06C152	Other digestive tract procedures apart from laparotomies
06C162, 06C163, 06C164	Operations on the esophagus, stomach and duodenum for malignant tumors
06C202, 06C204	Ulcer procedures on the esophagus, stomach and duodenum
06C211, 06C212, 06C213	Other laparotomy procedures on the digestive tract
06C221, 06C222, 06C223, 06C224	Procedures on the esophagus, stomach and duodenum for non-malignant conditions or ulcers
06C241, 06C242, 06C243, 06C244, 06C24J	Post-operative ventral cure
06C251, 06C252, 06C253, 06C254, 06C25J	Hernia repair procedures, excluding inguinal and crural hernias
07C04V	Cholecystectomies without exploration of the main bile duct
07C091, 07C092, 07C093, 07C094	Interventions on the liver, pancreas and portal or vena cava for malignant tumors
07C101, 07C102, 07C104	Interventions on the liver, pancreas and portal or vena cava for non-malignant diseases
07C112, 07C113	Bile diversions
07C121, 07C123	Other biliary tract procedures except isolated cholecystectomies
07C131, 07C132, 07C133, 07C134	Cholecystectomies without exploration of the main bile duct for acute conditions
07C141, 07C142, 07C14J	Cholecystectomies without exploration of the main bile duct, except for acute conditions

eTable 1c: Codes for pelvic surgeries

13C031, 13C032	Hysterectomies
13C051	Uteroannexal surgery for malignant tumors
13C071, 13C072	Interventions on the utero-annexal system for non-malignant conditions, other than tubal interruptions
13C081	Procedures on the vulva, vagina or cervix
13C141, 13C142, 13C143, 13C144	Pelvic exenteration, extended hysterectomy or vulvectomy for malignant tumors
13C151, 13C152	Pelvic exenteration, enlarged hysterectomy or vulvectomy for non-malignant conditions

eTable 1d: Codes for nervous system surgeries

01C032, 01C033, 01C034	Craniotomies for trauma
01C041, 01C042, 01C043, 01C044	Craniotomies without trauma
01C051, 01C052, 01C053, 01C054	Spine and spinal cord procedures for neurological conditions
01C081, 01C082, 01C083, 01C08J, 01C08V	Procedures on cranial or peripheral nerves and other nervous system procedures
08C271, 08C272, 08C273, 08C27V	Other spinal interventions
08C511, 08C512, 08C513, 08C514	Major spinal surgery for fractures, kyphosis and scoliosis
08C521, 08C522, 08C524	Other major spinal interventions

eTable 1e: Codes for ENT surgeries

08C281, 08C28J	Maxillofacial surgery

eTable 1f: Codes for orthopedic surgeries

05C121, 05C122, 05C123, 05C124, 05C12W	Lower limb amputations, except toes, for circulatory disorders
05C131, 05C132, 05C133, 05C134	Amputations for circulatory disorders of the upper limb or toes
08C022, 08C024	Multiple major knee and/or hip surgeries
08C05V, 08C05W	Hip and femur surgery, other than major surgery
08C062, 08C063	Amputations for musculoskeletal and connective tissue disorders
08C131, 08C133	Localized bone resections and/or removal of internal fixation hardware in the hip and femur
08C141, 08C142	Localized bone resections and/or removal of internal fixation hardware in sites other than the hip and femur
08C221, 08C222, 08C223, 08C224, 08C22Z	Joint prosthesis revision surgery
08C23W	Hip prostheses
08C241, 08C242, 08C243, 08C244, 08C24Z	Knee prostheses

© 2026 Bailly S et al. *JAMA Network Open*.

08C251, 08C252, 08C253	Shoulder prostheses
08C351, 08C352, 08C353, 08C35J	Operations on the arm, elbow and shoulder
08C391, 08C392, 08C393, 08C39J	Forearm surgery
08C401, 08C402	Arthroscopy of other sites
08C471, 08C472, 08C473	Hip prostheses for recent trauma
08C481, 08C482, 08C483, 08C484	Hip prostheses for conditions other than recent trauma
08C491, 08C492, 08C493	Hip and femur surgery for recent trauma
08C501, 08C502, 08C503	Hip and femur surgery, except for recent trauma
08C531, 08C532	Knee surgery for trauma
08C541, 08C543, 08C54J	Knee surgery for non-traumatic conditions
08C551, 08C552	Ankle and hindfoot fracture surgery
08C561, 08C562, 08C563, 08C564	Interventions for osteoarticular infections
08C571	Release of joints in the lower limb, with the exception of the hip and foot
08C581, 08C582, 08C58J	Shoulder arthroscopies
08C621, 08C622, 08C623, 08C624	Other procedures for osteoarticular infections

eTable 1g: Codes for urologic surgeries

11C021, 11C022, 11C023, 11C024	Kidney and ureter procedures and major bladder surgery for tumor disease
11C031, 11C032, 11C033, 11C034	Kidney and ureter procedures and major bladder surgery for non-tumor conditions
11C041, 11C043, 11C04J	Other bladder procedures excluding transurethral procedures
12C041, 12C042, 12C043, 12C044	Transurethral prostatectomy
12C111, 12C112, 12C113	Major pelvic surgery in men for malignant tumors
12C121, 12C122	Major pelvic surgery in men for non-malignant conditions

© 2026 Bailly S et al. *JAMA Network Open*.

eTable 2. Baseline Patient Characteristics According to PAP Status (Adherent vs no PAP or PAP Nonadherent)

Variable	All patients	No PAP	Non-adherent	Adherent	*P* value
N (%)	2,286	982 (43%)	576 (25.2%)	728 (31.8%)	
Age, Median [IQR]	58 [49-66]	57 [47-65]	57 [48-66]	60 [52-67]	<.01
Sex, % males	1472 (64.4)	587 (59.8)	381 (66.1)	504 (69.2)	<.01
BMI, Kg/m², Median [IQR]	30.4 [26.7-35.0]	28.8 [25.6-33.5]	30.8 [27-35.2]	31.5 [28-36.1]	<.01
ESS, Median [IQR]	10 [6-13]	9 [5-13]	10 [7-14]	10 [6-14]	<.01
Medical history					
CVD, n (%)	396 (17.3)	163 (16.6)	112 (19.4)	121 (16.6)	0.30
Stroke, n (%)	137 (6.3)	63 (6.7)	32 (6)	42 (6)	0.79
AF, n (%)	103 (4.8)	45 (4.9)	24 (4.5)	34 (4.9)	0.92
Heart failure, n (%)	119 (5.5)	51 (5.5)	26 (4.9)	42 (6.1)	0.68
CAD, n (%)	181 (8.3)	73 (7.8)	50 (9.2)	58 (8.3)	0.62
Hypertension, n (%)	971 (42.5)	378 (38.5)	247 (42.9)	346 (47.5)	<.01
Diabetes, n (%)	418 (18.32)	141 (14.4)	138 (24)	139 (19.1)	<.01
COPD, n (%)	305 (13.3)	124 (12.6)	78 (13.5)	103 (14.1)	0.65
SASHB, Median [IQR]	51.8 [24.0-100]	25.9 [14.2-50.8]	66.9 [40-118]	84.2 [52.9-149]	<.01
HB_{oxi}, Median [IQR]	56.3 [30.8-104]	34.5 [20-58.7]	70.8 [42.8-123]	89.2 [54.4-152]	<.01

Abbreviations: CV, cardiovascular; BMI, body mass index; CVD, cardiovascular diseases; AF, atrial fibrillation; CAD, coronary heart disease; COPD, chronic obstructive pulmonary diseases; SASHB, sleep apnea-specific hypoxic burden; PAP, positive airway pressure therapy; HB_{oxi}, simplified version of SASHB automatically derived from the single oximetry signal extracted from diagnostic sleep studies

© 2026 Bailly S et al. *JAMA Network Open*.

eTable 3. Duration Between Surgery and Complication Occurrence

Day after surgery	N (%)
Day 1	53 (66%)
Day 2 to Day 10	11 (14%)
Day 11 to Day 20	11 (14%)
Day 20 to Day 30	5 (6%)

© 2026 Bailly S et al. *JAMA Network Open*.

eTable 4. Univariable Logistic Regression Analyses of Factors Associated With the Primary Outcome.

Variable	Odds ratio [95 confidence interval]	*P* value
Age	1.04 [1.02-1.07]	<.0001
Men vs. Women	1.58 [0.95-2.61]	0.0775
Body mass index (Kg/m²)	1.01 [0.98-1.05]	0.4494
Tobacco consumption		0.6646
Former smokers	1.01 [0.62-1.63]	
Current smokers	0.75 [0.37-1.48]	.
No smokers	Reference	.
Living in couple vs. alone	0.80 [0.46-1.37]	0.4090
Professional situation		0.0108
Active	0.43 [0.25-0.76]	
Inactive	0.88 [0.45-1.71]	.
Retired	Reference	.
Socio-professional category		0.5118
Farmer	1.40 [0.46-4.28]	
Craftsman	1.33 [0.58-3.04]	.
Executive	1.43 [0.70-2.91]	.
Intermediate	0.62 [0.26-1.46]	.
Employee	1.18 [0.58-2.39]	.
Workers	Reference	.
Epworth Score	0.95 [0.91-1.00]	0.0393
Prevalent diseases		
History of cardiovascular disease before surgery	2.39 [1.47-3.87]	<.0001
Stroke	2.09 [1.01-4.29]	0.0448
Atrial fibrillation	1.11 [0.39-3.09]	0.8467
Heart failure	4.74 [2.61-8.64]	<.0001
Myocardial infarction	1.86 [0.96-3.60]	0.0633
Hypertension	1.44 [0.92-2.26]	0.1077
Diabetes	1.73 [1.04-2.87]	0.0319
Chronic obstructive pulmonary disease	1.40 [0.77-2.52]	0.2676
Type of diagnostic sleep study		0.0096
Polysomnography	Reference	.
Home sleep apnea testing	1.86 [1.16-2.99]	.
Apnea-hypopnea index (events/h)	1.02 [1.01-1.03]	<.0001
3% oxygen desaturation index (events/h)	1.02 [1.01-1.03]	<.0001
Sleep time with SpO_2<90% (%)	1.01 [1.00-1.02]	0.0030
Sleep apnea-specific hypoxic burden (%min/h)	1.004 [1.003-1.005]	<.0001
HB_{oxi}	1.004 [1.002-1.005]	<.0001
Duration between sleep exam and surgery	1.00 [1.00-1.00]	0.9790
Emergency admission before surgery	4.74 [2.92-7.70]	<.0001
Positive airway pressure (PAP) treatment status		
No PAP	Ref.	0.0201
PAP non-adherent (termination or <4h/night)	1.01 [0.54 -1.89]	.
PAP adherent (≥4h/night)	1.89 [1.14-3.14]	.

Abbreviations: PAP, positive airway pressure therapy; HB_{oxi}, simplified version of SASHB automatically derived from the single oximetry signal extracted from diagnostic sleep studies

© 2026 Bailly S et al. *JAMA Network Open*.

eTable 5. Association of Sleep Apnea-Specific Hypoxic Burden (SASHB) with the Primary Composite Outcome (All-Cause Mortality, Stroke, Atrial Fibrillation, Heart Failure, Myocardial Infarction, and Venous Thrombo-Embolism) Within 30 Days of Surgery, in the Entire Population and in Patient Subgroups.

		N events	N	Percent	Adjusted OR [95%CI]	P value
**All patients N=2,286**						
	1. SASHB 0-32	12	762	1.6	Ref.	0.0086
	2. SASHB 32-80	24	762	3.1	1.759 [0.862; 3.591]	
	3. SASHB >80	44	762	5.8	2.792 [1.419; 5.491]	
**Age - Pi=0.9998**						
<58 years N=1,107	1. SASHB 0-32	6	479	1.2	Ref.	0.9998
	2. SASHB 32-80	8	343	2.3	1.569 [0.528; 4.665]	
	3. SASHB >80	12	285	4.2	2.451 [0.868; 6.922]	
≥58 years N=1,179	1. SASHB 0-32	6	283	2.1	Ref.	0.0122
	2. SASHB 32-80	16	419	3.8	1.838 [0.702; 4.814]	
	3. SASHB >80	32	477	6.7	3.014 [1.210; 7.509]	
**Sex – Pi=0.3729**						
Men N=1,472	1. SASHB 0-32	6	436	1.4	Ref.	
	2. SASHB 32-80	18	464	3.9	2.596 [1.003; 6.721]	0.0165
	3. SASHB >80	35	572	6.1	3.377 [1.366; 8.346]	
Women N=814	1. SASHB 0-32	6	326	1.8	Ref.	
	2. SASHB 32-80	6	298	2.0	0.929 [0.290; 2.973]	0.0610
	3. SASHB >80	9	190	4.7	2.300 [0.772; 6.854]	
**History of CVD - Pi=0.4211**						
Yes N=396	1. SASHB 0-32	1	106	0.9	Ref.	
	2. SASHB 32-80	8	133	6.0	7.588 [0.898; 64.10]	0.0403
	3. SASHB >80	17	156	10.9	13.63 [1.648;112.8]	
No N=1,890	1. SASHB 0-32	11	655	1.7	Ref.	
	2. SASHB 32-80	16	629	2.5	1.276 [0.579; 2.811]	0.1244
	3. SASHB >80	27	606	4.5	2.037 [0.970; 4.274]	
**Type of sleep study - Pi=0.1704**						
PSG N=1,101	1. SASHB 0-32	4	444	0.9	Ref.	
	2. SASHB 32-80	13	386	3.4	3.449 [1.063; 11.19]	0.1091
	3. SASHB >80	10	271	3.7	3.150 [0.888;11.17]	
HSAT N=1,185	1. SASHB 0-32	8	318	2.5	Ref.	
	2. SASHB 32-80	11	376	2.9	1.077 [0.423; 2.742]	0.0309
	3. SASHB >80	34	491	6.9	2.306 [1.032; 5.152]	
**Time from diagnosis to surgery - Pi=0.3350**						
< 4.5 years N=1,143	1. SASHB 0-32	4	367	1.1	Ref.	
	2. SASHB 32-80	10	372	2.7	1.990 [0.600; 6.600]	0.0406
	3. SASHB >80	25	404	6.2	3.704 [1.217; 11.27]	
≥4.5 years	1. SASHB 0-32	8	395	2.0	Ref.	

© 2026 Bailly S et al. *JAMA Network Open*.

N=1,143	2. SASHB 32-80	14	390	3.6	1.548 [0.626; 3.833]	0.2198
	3. SASHB >80	19	358	5.3	2.174 [0.899; 5.255]	
**PAP status - Pi=0.8931**						
Adherent N=728	1. SASHB 0-32	1	76	1.3	Ref.	
	2. SASHB 32-80	9	260	3.5	2.383 [0.292; 19.46]	0.3235
	3. SASHB >80	27	392	6.9	3.633 [0.471; 28.04]	
No PAP or not Adherent N=1,558	1. SASHB 0-32	11	686	1.6	Ref.	
	2. SASHB 32-80	15	502	3.0	1.570 [0.701; 3.519]	0.1436
	3. SASHB >80	17	370	4.6	2.251 [1.003; 5.050]	

Abbreviations: CVD, cardiovascular diseases; PSG, polysomnography; HSAT, home sleep apnea testing; PAP, positive airway pressure therapy.

*Models were adjusted for age, sex, history of CVD, COPD emergency admission before surgery and tobacco consumption.

© 2026 Bailly S et al. *JAMA Network Open*.
