# Supplementary material for: Sleep Apnea-Specific Hypoxic Burden and Postoperative Outcomes of Major Noncardiothoracic Surgery
Source: JAMA Netw Open. 2026 Feb 24;9(2):e260006. doi: 10.1001/jamanetworkopen.2026.0006 (PMC12933281; doi:10.1001/jamanetworkopen.2026.0006)
Supplement: Supplement 2. — Data Sharing Statement [file jamanetwopen-e260006-s002.pdf]

Data Sharing Statement

Bailly. Sleep Apnea-Specific Hypoxic Burden and Postoperative Outcomes of Major Noncardiothoracic Surgery. *JAMA Netw Open*. Published February 24, 2026. doi:10.1001/jamanetworkopen.2026.0006

Data
Data available: No

Additional Information
Explanation for why data not available: Data are from National Health Insurance database and data sharing is not allowed.
